# Remembering the forgotten child: the role of immune checkpoint inhibition in patients with human immunod eficiency virus and cancer

**DOI:** 10.1186/s40425-019-0618-9

**Published:** 2019-05-22

**Authors:** Jacob J. Adashek, Pedro Nazareth Aguiar Junior, Natalie Galanina, Razelle Kurzrock

**Affiliations:** 1 Department of Internal Medicine, University of South Florida, H. Lee Moffitt Cancer Center & Research Institute, Tampa, FL USA; 2 Faculdade de Medicina do ABC, Santo André, Brazil; 3 Américas Centro de Oncologia Integrado, São Paulo, Brazil; 4 Center for Personalized Cancer Therapy and Clinical Trials Office, Division of Hematology and Oncology, Clinical Science, Department of Medicine, University of California San Diego Moores Cancer Center, 3855 Health Sciences Drive, La Jolla, CA 92093 USA

**Keywords:** Human immunodeficiency virus, Immunotherapy, Cancer clinical trials, Immune checkpoint inhibitors

## Abstract

Patients with human immunodeficiency virus (HIV) infection have a high risk of developing virally-mediated cancers. These tumors have several features that could make them vulnerable to immune checkpoint inhibitors (ICIs) including, but not limited to, increased expression of the CTLA-4 and PD-1 checkpoints on their CD4+ T cells. Even so, HIV-positive patients are generally excluded from immunotherapy cancer clinical trials due to safety concerns. Hence, only case series have been published regarding HIV-positive patients with cancer who received ICIs, but these reports of individuals with a variety of malignancies demonstrate that ICIs have significant activity, exceeding a 65% objective response rate in Kaposi sarcoma. Furthermore, high-grade immune toxicities occurred in fewer than 10% of treated patients. The existing data suggest that the underlying biologic mechanisms that mediate development of cancer in HIV-infected patients should render them susceptible to ICI treatment. Preliminary, albeit limited, clinical experience indicates that checkpoint blockade is both safe and efficacious in this setting. Additional clinical trials that include HIV-positive patients with cancer are urgently needed.

## Background

Approximately 15–20% of all cancers can be attributed to a viral antecedent [1]. This number is amplified by the immunodeficiency that ensues following human immunodeficiency virus (HIV) infection, which creates the immunologic milieu conducive to virally-induced oncogenesis. Examples of virus-induced cancers that propagate in the setting of HIV-infection and immunodeficiency as well as immune mechanisms are presented in Table 1 [1–13]. Treatment of HIV-positive cancer patients with traditional cytotoxic therapy can further exacerbate the already compromised immune status as well as create potential drug-drug interaction with the anti-retroviral therapy (ART). Hence, the development of novel therapeutics to expand the anti-neoplastic armamentarium for these patients is an area of unmet clinical need.

**Table 1:** Examples of virally associated neoplasms reported in HIV-infected individuals, response to checkpoint blockade and mechanisms of action as well as mechanisms of action

Cancer	Virus	Immunotherapy	Response Rate (n/total n) (X%)	Common Side Effects
Anal Cancer	Human Papilloma Virus	Nivolumab	1/2; 50% [2]	Anemia, fatigue, rash, hypothyroidism
Burkitt’s lymphoma	Epstein Barr Virus	Not reported	Not reported	Not reported
Central nervous system lymphoma	Epstein Barr Virus	Not reported	Not reported	Not reported
Cervical Cancer	Human Papilloma Virus	Not reported	Not reported	Not reported
Hodgkin disease	Epstein Barr Virus	Nivolumab	1/1; 100% [3]	Not reported
Kaposi Sarcoma	Human Herpes Virus-8	Nivolumab or pembrolizumab	6/9; 67% [4]	Fatigue, gastrointestinal discomfort, pruritis, onycholysis
Kaposi sarcoma-associated herpesvirus multicentric Castleman disease	Human Herpes Virus-8	Not reported	Not reported	Not reported
Merkel Cell Carcinoma	Merkel Cell Polyomavirus	pembrolizumab or avelumab	2/2; 100% [5, 6]	Pneumonitis
Nasopharyngeal Carcinoma	Epstein Barr Virus	Not reported	Not reported	Not reported
Penile Cancer	Human Papilloma Virus	Not reported	Not reported	Not reported
Plasmablastic lymphoma	Epstein Barr Virus	Not reported	Not reported	Not reported
Primary effusion lymphoma	Human Herpes Virus-8	Not reported	Not reported	Not reported
Vulvar Cancer	Human Papilloma Virus	Not reported	Not reported	Not reported

Biologic mechanisms that are amenable to immune checkpoint blockade associated with cancers in HIV-infected patients

Viral antigens presented by host cells are recognized as foreign [1]

CD4+ T cells in HIV-positive patients have increased expression of the checkpoints CTLA-4 and PD-1^9,11^

The host DNA damage response is impaired in virally-mediated cancers [12]

APOBEC-related mutagenesis is associated with viruses and increases neopeptide hydrophobicity/immunogenicity and correlates with higher levels of PD-L1 expression [7, 8]

### Relationship between HIV and immune checkpoint molecules

The therapeutic landscape for malignancies is rapidly evolving with the advent of immune checkpoint inhibitors (ICI), most notably programmed-cell death (ligand)-1 (PD-(L)1) and cytotoxic T-lymphocyte-associated protein 4 (CTLA-4) inhibitors. Taking into account that one of the hallmarks of cancer is its innate ability to evade the immune system, ICIs may hold transformative potential owing to their ability to block the suppressive immune signals produced by tumor cells. These agents have impressive clinical activity in a broad array of both solid and hematologic malignancies, including patients with advanced, refractory disease. To date, seven checkpoint inhibitors have been approved by the Food and Drug Administration (FDA).

Despite the encouraging results with ICIs in multiple cancer types, there is a paucity of data regarding the use of these agents in patients with HIV-associated malignancies because these patients are often excluded from clinical trials. Yet, patients living with HIV have a significantly higher incidence of cancer including non-Hodgkin lymphoma (~ 21% of cancers in HIV-infected people), Kaposi sarcoma (~ 12%), lung cancer (~ 11%), anal/cervical cancer (~ 10%), as well as other tumor types such as colorectal (~ 5%), oral/pharyngeal (4%), and others (NCCN guidelines version 2.2019 AIDS related Kaposi sarcoma (https://www.nccn.org/professionals/physician_gls/pdf/kaposi.pdf)(https://www.cancer.gov/about-cancer/causes-prevention/risk/infectious-agents/hiv-fact-sheet).

Importantly, patients with rampant HIV infections – high viral loads in the absence of being on antiretroviral therapy (ART) – have more expression of CTLA-4 on their CD4+ T cells when compared to those of healthy controls (Table 1). Additionally, CTLA-4 levels are inversely related to total CD4+ T cell population and directly related to HIV viral load and cancer progression [11]. This same patient population also expresses higher levels of PD-1 on their CD4+ T cells when compared to those of healthy controls and this is associated with T cell exhaustion; further, similar to CTLA-4, PD-1 levels are also related to HIV viral load and cancer progression [9, 10]. The dual changes in checkpoint cell surface molecules in patients who are infected with HIV and have cancer could be exploited in their treatment as is being explored in a clinical trial of ipilimumab with nivolumab in HIV-associated solid tumors and lymphoma (NCT02408861) as well as with pembrolizumab monotherapy in HIV and various cancers (NCT02595866) (clinicaltrials.gov). HIV evades the immune response by promoting a state of immune exhaustion, which is similar to the mechanism of how cancers with upregulated PD-L1/PD-1 axis and/or CTLA-4 expression elude immune eradication [9, 10, 13]. Therefore, in theory, ICIs may be beneficial to both the HIV infection and to cancer.

### Virus-induced cancers: immune and mutational landscape and Neoantigen immunogenicity

Mechanistically, not only do viral infections lead to an increase in expression of the checkpoint cell surface molecules CTLA-4 and PD-1, they also subvert the DNA damage response within the host cell DNA. Indeed, DNA viruses (EBV, HHV-8, HPV, etc.) thrive by inserting their viral genome into the genome of the host cell and subsequently hijacking the host cell replicative enzymes [12]. The host DNA damage response that should be activated in response to the replicating viral DNA or the virally-stimulated cellular transition from a quiescent to mitotic/cell cycle state, induced by viruses in order to facilitate replication, is attenuated by specific proteins expressed by the DNA tumor viruses. Virally-induced cancers may also have distinct mutational portfolios and metabolic patterns that can impact immune response and prognosis. For instance, HPV-associated squamous tumors of the head and neck may harbor more *PIK3CA* alterations whereas non-HPV tumors may have *TP53* and cyclin pathway (*CDKN2A* and *CCND1)* alterations. Finally, molecular editing mechanisms mediated by apolipoprotein B mRNA editing enzyme catalytic polypeptide-like (APOBEC), a family of evolutionarily conserved cytidine deaminases involved in DNA and mRNA editing that are upregulated with viral infection leading to inactivation of viral genomes, may be relevant. These upregulated enzymes constitute a crucial part of mammalian innate immunity and are also a major source of mutations in multiple cancer types. Relevantly, APOBEC-related mutagenesis increases neoantigen hydrophobicity, a key feature of immunogenicity [7]. Cancers with upregulated APOBEC show high levels of PD-L1 expression that presumably enable the cancer to evade the immune system and survive in light of the immunogenic mutation-related peptides induced by APOBEC [8]. Hence, not surprisingly, PD-1/PD-L1 ICIs are associated with high response rates in human cancers that bear APOBEC mutational gene expression patterns [7, 8]. The production of mutations that result in immunogenic neoantigens or presentation of the viral antigens themselves may also explain the fact that malignancies such as virus-related Merkel cell carcinoma and Kaposi sarcoma respond well to ICIs, despite a low tumor mutational burden, the latter usually being associated with a poor response to these immunotherapeutics [4, 14, 15].

### Activity of immune checkpoint inhibitors among HIV-positive patients with cancers

There have been only a small number of case series on the use of ICIs in HIV-positive patients. A recent review that included all studies published (73 HIV-patients suffering from several primary tumors treated with either PD-1, CTLA-4, or both inhibitors) found a response rate of 67% for Kaposi sarcoma, 30% for non–small cell lung cancer, and 27% for melanoma; in addition, activity including complete responses was seen in Merkel cell carcinoma and in Hodgkin lymphoma [4, 16]. Importantly, only 9% of individuals reported greater than or equal to grade 3 immune-related toxicities, most of which occurred in patients who received ipilimumab as part of their regimen [16]. HIV remained suppressed in 93% of patients with available data and undetectable viral load and, overall, CD4+ counts increased [4, 16]. The low numbers of patients in the dataset reflects the practice of exclusion of HIV-positive patients from the majority of studies. However, the activity of ICIs in these reports and their lack of toxicity suggest that additional trials are needed.

## Discussion

Virally-induced cancers suppress the host DNA damage response machinery and activate enzymes such as APOBEC that mutate both the virus and the host genome; in the latter, the mutations have high hydrophobicity, a feature associated with neopeptide immunogenicity for T cells [7]. Viral antigens themselves may also be presented by the host cells and would presumably be recognized as foreign by the immune system. Upregulation of PD-L1 may accompany these changes and, since this ligand neutralizes the immune system, the tumors can proliferate without immune recognition [7, 8]. Patients with HIV infection and cancer also demonstrate high expression of CTLA-4 and PD-1 on their lymphocytes [9, 11]. All of these changes make these tumors hypothetically vulnerable to ICI treatment. Despite this potential activity, there have been several concerns that have led to the exclusion of HIV-infected individuals with cancer from trials utilizing ICIs. First, ICI-activated lymphocytes may not work properly in HIV-infected patients due to their immunodeficiency. However, the absolute decrease in the number of CD4+ T cells is overcome with the use of ART, and, in general, in the small number of patients reported, CD4+ counts have tended to increase after ICI treatment [4, 16]. Another concern might be the potential increased risk of immune-related complications after ICIs in patients with a dysregulated immune system. However, only 9% of patients in a review of 73 treated individuals developed high-grade immune-related toxicities [16]. Therefore, ICIs for the therapy of advanced-stage malignancies in patients with HIV infection was associated with no new safety signals. HIV load remained suppressed in most patients. Furthermore, anecdotally, patients with high HIV load can respond without undue toxicity [4, 16, 17].

There might also be unease regarding potential reactivation of viruses such as hepatitis B virus, with anecdotal reports of this occurrence in HIV-infected patients treated with ICIs. However, the risk of hepatitis B virus reactivation is probably a more serious concern for patients treated with conventional myelosuppressive chemotherapy. Indeed, recent guidelines recommend preemptive antiviral therapy for hepatitis B surface antigen-positive patients undergoing chemotherapy, irrespective of their baseline viral load or HIV status. Finally, many drugs can interact with ART. The interaction between ART and ICIs will need better definition though, as mentioned, to date, it appears that the viral load remained suppressed in the vast majority of individuals with undetectable viral loads treated ICIs [16]. Overall, various case reports and small series have served as rationale for the use of ICI in HIV-positive patients with varying malignancies (Table 1). For instance, favorable responses without toxicity in HIV-associated Kaposi sarcoma [4] provide a rationale for future studies.

## Conclusion

HIV-infected patients are underrepresented in ICI clinical trials, despite reports that have clearly demonstrated promising activity and excellent safety with ICIs among different advanced malignancies [4, 16]. There are clinical trials with checkpoint blockade that include HIV-positive patients with well-controlled disease [18]; however, it is likely that only a minority of patients on such trials are actually HIV-positive, and therefore learning about their outcomes from single trials may be difficult. Additional studies designed for HIV-positive patients with malignancies are urgently needed. Furthermore, based on the mechanistic likelihood of ICI response in cancers that occur in HIV-infected patients and the substantial efficacy seen in the small series to date, these patients should not be excluded from immunotherapy clinical trials.

## Abbreviations

APOBEC: apolipoprotein B mRNA editing enzyme, catalytic polypeptide-like
ART: anti-retroviral therapy
ART: Antiretroviral therapy
CTLA-4: cytotoxic T-lymphocyte-associated protein 4
FDA: Food and Drug Administration
HIV: human immunodeficiency virus
ICIs: immune checkpoint inhibitors
PD-(L)1: programmed-cell death (ligand)-1
